# miR-192 Directly Binds and Regulates Dicer1 Expression in Neuroblastoma

**DOI:** 10.1371/journal.pone.0078713

**Published:** 2013-11-01

**Authors:** Galina Feinberg-Gorenshtein, Avital Guedj, Keren Shichrur, Marta Jeison, Drorit Luria, Yona Kodman, Shifra Ash, Meora Feinmesser, Liat Edry, Noam Shomron, Abraham Weizman, Isaac Yaniv, Smadar Avigad

**Affiliations:** 1 Molecular Oncology, Felsenstein Medical Research Center, Petah Tikva, Israel; 2 Sackler Faculty of Medicine, Tel Aviv University, Tel Aviv, Israel; 3 Pediatric Hematology Oncology, Schneider Children's Medical Center of Israel, Petah Tikva, Israel; 4 Institute of Pathology, Rabin Medical Center, Petah Tikva, Israel; 5 Department of Cell and Developmental Biology, Sackler Faculty of Medicine, Tel Aviv University, Tel Aviv, Israel; French National Center for Scientific Research - Institut de biologie moléculaire et cellulaire, France

## Abstract

Neuroblastoma (NB) arises from the embryonic neural crest and is the most common extracranial solid tumor in children under 5 years of age. Reduced expression of Dicer1 has recently been shown to be in correlation with poor prognosis in NB patients. This study aimed to investigate the mechanisms that could lead to the down-regulation of Dicer1 in neuroblastoma. We used computational prediction to identify potential miRs down-regulating Dicer1 in neuroblastoma. One of the miRs that were predicted to target Dicer1 was miR-192. We measured the levels of miR-192 in 43 primary tumors using real time PCR. Following the silencing of miR-192, the levels of dicer1 cell viability, cell proliferation and migration capability were analyzed. Multivariate analysis identified miR-192 as an independent prognostic marker for relapse in neuroblastoma patients (p=0.04). We were able to show through a dual luciferase assay and side-directed mutational analysis that miR-192 directly binds the 3' UTR of Dicer1 on positions 1232-1238 and 2282-2288. An increase in cell viability, proliferation and migration rates were evident in NB cells transfected with miR-192-mimic. Yet, there was a significant decrease in proliferation when NB cells were transfected with an miR-192-inhibitor We suggest that miR-192 might be a key player in NB by regulating Dicer1 expression.

## Introduction

Neuroblastoma (NB) arises from the embryonic neural crest and is the most common extracranial solid tumor in children under 5 years of age [1]. NB is characterized by a wide range of clinical behaviors, from spontaneous regression to rapid progression with a fatal outcome [1]. The clinical heterogeneity of NB has been reported to be associated with a variety of biological and molecular features: aged 18 months or more, advanced stages 3 and 4, adrenal primary site, MYCN amplification (MYCNA) and diploid or tetraploid DNA index (DI) are considered adverse indicators [1]. 

MicroRNAs (miRs) are non-coding, single-stranded 18-24 nucleotide RNA molecules that base-pair with target mRNAs and negatively regulate their stability and translation efficiency [2,3]. More than 50% of miRs are located in cancer-associated genomic regions or in fragile sites, suggesting that miRs play an important role in pathogenesis of human cancers [4]. Dicer1 together with Drosha, catalyze the sequential cleavage of miR maturation [5]. 

Recently, it has been demonstarted that reduced expression of Dicer1 correlated with a poor prognosis in NB patients [6]. We were intersted in exploring the mechanisms leading to the down-regulation of Dicer1 in NB. We hereby report that miR-192 represents one of previously unrecognized miRs that regulate Dicer1 expression in NB.

## Materials and Methods

### Patient Samples

The samples were obtained from the tissue bank of the Pediatric Hematology-Oncology Department at the Schneider Children’s Medical Center of Israel, Helsinky approval 0012-08-RMC. The Institutional Review Board (IRB) Rabin Medical Center, Petah Tikva, Israel and the National Ethical Committee, Ministry of Health, Jerusalem, Israel, approved the research project. Obtaining informed consent for this study was specifically waived by the IRB. Each tumor specimen was assayed for tumor cell content, histopathology, DI and MYCNA status. 

The cohort consisted of primary tumors obtained from 69 patients (not all samples were available for evaluation of all assays). Forty four (64%) patients were above 18 months of age. The site of the primary tumor was adrenal in 28% of the patients. MYCNA was identified in 11 (16%) tumors. High risk (HR) NB is defined as NB stage 3 with MYCNA or stage 4, diagnosed according to the International Neuroblastoma staging system (INSS) criteria. Thirty six (52%) patien**t**s were defined as HR. A DI of diploid/ tetradiploid was detected in 38 tumors (60%). Relapse occurred in 18 (33%) patients. The median follow-up was 110 months (range, 4-289).

### Cell lines

NB cell lines (SHEP and NUB6) were cultured according to the ATCC growth recommendations. All ATCC cell lines undergo authentication tests during the accessioning process.

### Bioinformatics

We searched three miR databases: miRBase Sequence Database (http://microrna.sanger.ac.uk/sequences), Target scan (http://www.targetscan.org/) and TargetRank (http://genes.mit.edu/targetrank/) for miRs that target Dicer1, their sequences and their chromosome localization.

### RNA purification

RNA was purified from tissues and cell lines using miRNeasy Mini Kit (Qiagen, Valincia, CA, USA) and from paraffin embedded (FFPE) tissue sections (3-5 sections of 5μm thicknesses) using miRNeasy FFPE kit (Qiagen, Valincia, CA, USA), according to the manufacturer’s protocol. 

For Dicer1 expression analysis we extracted total RNA using RNeasy Mini Kit (Qiagen, Valincia, CA, USA).

### cDNA synthesis and real time quantitative PCR (RQ-PCR)

cDNA synthesis and RQ-PCR amplification of miR-103, miR-124, miR-192, miR-612 and mir-125b-1 were performed in 43 NB samples using miScript Reverse Transcription Kit (Qiagen, Valincia, CA, USA) and miScript SYBR® Green PCR Kit (Qiagen, Valincia, CA, USA), respectively, according to the manufacturer’s protocol. RQ-PCR reactions were performed in duplicates and the relative expression levels of studied miRs were normalized using the 5S snRNA endogenous control, using the ΔCt method. 

We used ImProm-II Reverse Transcription kit (Promega Corporation, Madison, WI, USA) for cDNA synthesis of Dicer1 and the cDNA was cleaned by DNA Clean & Concentrator (Zymo Research Corporation, Orange CA, USA).

RQ-PCR amplification was performed in 47 NB patients using LightCycler FastStart DNA Master SYBR Green I (Roche Diagnostics GmbH, Manheim, Germany).

The primer pairs for Dicer1 **a**re described elsewhere [7]. The primer pairs for the housekeeping gene, GAPDH, were: forward: 

5'-CAACAGCCTCAAGATCATCAGC-3' and reverse: 

5'- CTCATGACCACAGTCCATGCCA- 3'

The expression levels of all samples were measured in the LightCycler 480 (Roche Diagnostics GmbH, Manheim, Germany).

### Multiplex Ligation-dependent Probe Amplification (MLPA)

MLPA analysis was performed in 40 NB samples using the NB specific SALSA MLPA probe consisting of three kits (P251/P252/P253, MRC-Holland), covering 115 loci within the ten chromosomal regions of the highest interest (plus reference loci) within a given sample. Tests were performed according to the manufacturer's instructions, and data was analyzed with MLPAVizard, a software specially developed for the NB kit and kindly provided by Prof. Ambros and the Austrian Research Center GmbH, Vienna, Austria. 

Genomic DNA was extracted from BM samples at diagnosis using Gentra Puregene Blood Kit (Qiagen, Valincia, CA, USA).

### miR transfections

Cells were seeded to 60-70% confluence in a 6-well plate (Cellstar, Washington, DC, USA) and were transfected with 0.5nM or 1nM miR-192 mimics or inhibitors (Qiagen, Valincia, CA, USA), using INTERFERin transfection reagent (Polyplus-transfection, New York, USA), according to the manufacturer’s protocol, in at least three independent experiments. The cells were cotransfected with 0.5 nmol and 1 nmol Allstars negative control siRNA (Qiagen, Valincia, CA, USA).

### Western blotting

Amounts of 40 mg/ml protein from each lysate were separated on 10% Bis-Tris gel for 1.5h in MOPS running buffer and transferred after 1.5h. The membrane was incubated for 20 min in 5% Blot-QuickBlocker (Calbiochem, San Diego, California) in Tween-Tris-buffered salt (TTBS). Hybridization was performed with Dicer1 (1:500) (Anti-Dicer, Rabbit mAb, Qiagen, Valincia, CA, USA), Tubulin (1:1000) (Anti-Tubulin, Mouse mAb, Sigma, Saint Louis, Missouri, USA), and secondary antibody (1:10000) (Sigma, Saint Louis, Missouri, USA). 

### Site-directed mutagenesis

**Figure 1:**
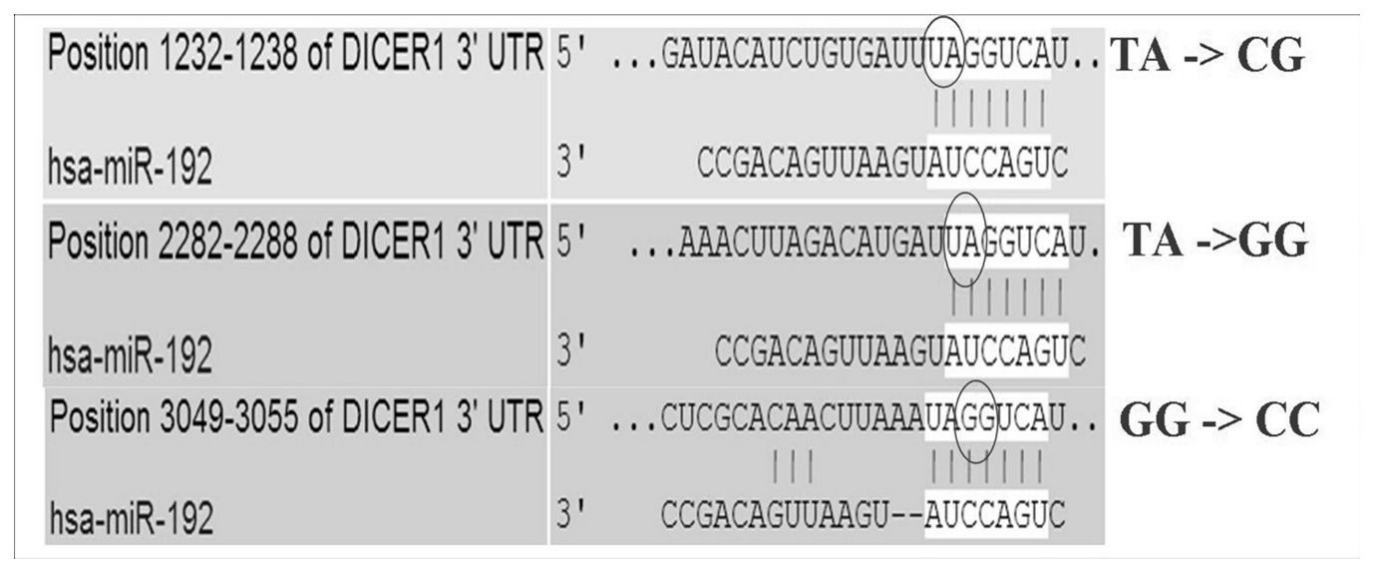
Site-directed mutagenesis of miR-192 binding sites on 3' UTR of Dicer1. Three mutations were introduced into the three potential binding sites of miR-192 on 3' UTR of pI DICER1 using Multi Site-Drected Mutagenesis Kit . The nucleotides that were mutated are circled and were changed to the nucleotides in bold.

We received Dicer1 plasmid ("Addgene plasmid 21649") from the non-profit plasmid repository Addgene (http://www.addgene.org) [8]. We created four new plasmids by using QuikChange Lightning Multi Site-Directed Mutagenesis Kit (Stratagene, Santa Clara, CA, USA) according to the manufacturer's instructions (Figure 1). The first plasmid contained mutations in all three BSs (ALL MUT).The second plasmid contained two mutations in BS2 (position 2282-2288; the TA nucleotides were changed to the GG nucleotides) and BS3 (position 3049-3055; the GG nucleotides were changed to the CC nucleotides); the third plasmid consisted of mutations in BS1 (position 1232-1238; the TA nucleotides were changed to the CG nucleotides);) and BS3 . The fourth plasmid included mutations in BS1 and BS2.

Primers were designed using QuikChange Primer Design Program (www.agilent.com/genomics/qcpd). Plasmids were extracted by HiSpeed Plasmid Midi Kit (Qiagen, Valincia, CA, USA), and mutant inserts were confirmed by DNA sequencing.

### Dual - luciferase assay

A total of 1 × 10^5^ cells per well were plated in 24 well plates and were transfected the following day using two different methods:

1 Overexpression or silencing of miR-192 by transfecting the cells with miR-192 mimic or inhibitor, respectively. As a negative control we used miR with scramble sequence (Qiagen, Valincia, CA, USA).
2 Overexpression of miR-192 by transfecting the cells with miR-192-vec (pMSCV-Blasticidin vector under the control of a CMV promoter) or miR-Vec-Ctrl (control). Both vectors were kindly provided by Dr. Reuven Agami (The Netherlands Cancer Institute). miR-Vec-Ctrl was made by deleting the MCS and the PGK-promoter from pMSCV-Blast, followed by the insertion of the CMV promoter from pcDNA-3.1+ and a stuffer DNA.

In both sets of experiments, the cells were cotransfected with pIS1 DICER1 long UTR or pIS1 DICER1 long UTR MUT (plasmid with mutations in all three potential binding sites), expressing Renilla Luciferase gene, using jetPRIME™ transfection reagent (Polyplus-transfection, New York, USA). The plasmid PGL3 (Promega Corporation, Madison, WI, USA), expressing the firefly luciferase gene, was used as an internal control for each sample. After 48 hours, cells were assayed for luciferase activities by Dual-Luciferase® reporter assay system (Promega Corporation, Madison, WI, USA), using a luminometer Berthold Plate Reader (Berthold Technologies). The Relative Light Unit (RLU) was calculated as the ratio of the expression value of renilla luciferase to that of firefly luciferase.

### Cell viability and proliferation

The NUB6 and SHEP cell lines were cultured in 10cc plates overnight and then transfected with 0.5 nM or 1 nM miR-192 mimic or miR-192 inhibitor and a negative control for 48 hours. Cell viability was determined by using the Trypan–blue staining assay. Data were plotted as means ± SD of three separate experiments.

Cell proliferation was detected using the XTT kit (Biological Industries Ltd., Kibbutz Beit Haemek, Israel) according to the manufacturer's instructions. The absorbance of the samples was measured with a spectrophotometer at a wavelength of 450-500 nanometers. In order to measure reference absorbance (to measure non-specific readings), a wavelength of 630-690 nanometer was used.

### Migration assay

Tumor cell migration was measured using the InnoCyte™ Cell Migration Assay (Calbiochem, Darmstadt, Germany). 2.5x10^5^ NUB6 cells following 24 hr of transfection with miR-192 mimic, miR-192 inhibitor or scramble miR sequence, were incubated in triplicate wells of the upper chamber and migrated towards the lower chamber, containing medium with and without 30% FCS, for 24h at 37°C. The results were presented as an average of 3 experiments; each was run in triplicates and analyzed using a standard fluorescent plate reader at 485/520 nm.

### Statistics

miRs and Dicer1 gene expression levels were dichotomized into high and low expression within each cohort, using the median as cutoff. Fisher's exact test was used for detecting a correlation between miR expression and clinical parameters such as: stage, MYCNA, risk, DI and primary tumor location. Progression free survival (PFS) was estimated by Kaplan Meier analysis by high or low miR expression levels. Cox regression analysis for relapse was performed using the following variants: miR expression levels, age, stage and MYCN status.

The effects of miR-192 on NB cell viability and proliferation was calculated using the mean and the standard deviation of at least three independent experiments. Analysis of variance was performed with Student’s t test using Excel software for the correlation between 14q deletion and Dicer1 expression level. A *p* value of 0.05 or less was considered statistically significant.

## Results

### Expression analysis of Dicer1 in NB tumors

**Figure 2:**
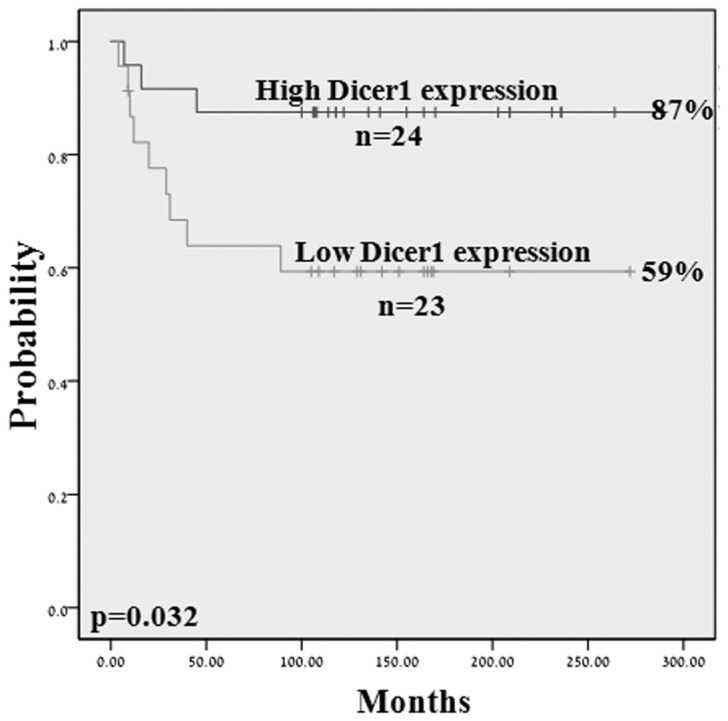
Kaplan Meier analysis by Dicer1 expression. Kaplan Meier analysis for PFS by Dicer1 expression (n=47): high and low expression levels of Dicer1 were determined as above (n=24) or below (n=23) the median expression level.

Expression levels of Dicer1 mRNA were analyzed in a cohort of 47 NB samples. We identified a significant correlation between PFS and Dicer1 expression at 10 years: patients with high Dicer1 expression levels had 87% PFS versus 59% in patients with low Dicer1 expression (p=0.032, Figure 2). No significant correlation was identified between Dicer1 expression levels and clinical parameters.

Chromosomal deletion might be a mechanism for the down-regulation of Dicer1 . Since Dicer1 is located on 14q32.13, we evaluated the deletion of chromosome 14q32 by MLPA.

### MLPA analysis

MLPA analysis was performed in 40 samples. A deletion was identified in only two cases. Thus, the 14q32 deletion is not a frequent abnormality in our cohort and could not play a role in the down-regulation of Dicer1 in NB.

### Expression analysis of miRs in NB tumors

An additional mechanism for the down-regulation of Dicer1 could be by miR regulation. We used computational prediction to identify potential miRs that down-regulate Dicer1 in NB. Of the predicted miRs we decided to focus on 5 miRs: miR-103, miR-124, mir-125b-1, miR-612 and miR-192. 

**Figure 3:**
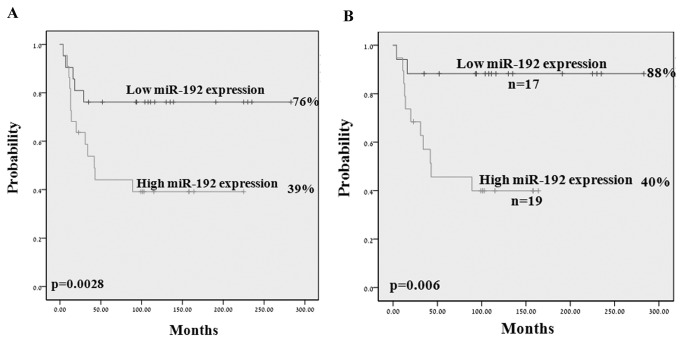
Kaplan Meier analysis by miR-192 expression. Kaplan Meier analysis for PFS by miR-192 expression: high and low miR-192 expression levels were determined as above or below the median expression level and were analyzed in (A) a whole cohort (n=43) and in (B) a cohort following exclusion of MYCNA (n= 36) .

We analyzed the relative expression levels of the miRs in a cohort of 43 NB primary tumors. We did not detect a significant correlation between clinical parameters or outcome and expression levels of miR-103, miR-124, miR-612 or mir-125b-1. No significant association between miR-192 expression levels and clinical markers was identified. However, a significant correlation was detected with outcome at 10 years. Patients with low miR-192 expression levels had 76 % PFS versus 39% in patients expressing high miR-192 levels (p= 0.028; Figure 3A). Following the exclusion of MYCNA patients (n=36), the correlation became even stronger: patients with low miR-192 expression levels had 88 % PFS versus 40 % in patients with high miR-192 (p= 0.006; Figure 3B). Multivariate analysis including the variants stage, MYCN, HR group and miR-192, identified miR-192 as an independent prognostic marker for relapse (p= 0.04), in addition to the HR group (Table 1). A patient expressing high levels of miR-192 had a 2.9-fold increased risk to relapse. 

**Table 1:** Univariate and multivariate Cox regression analyses for relapse.

**Multivariate**	**Univariate**	**Variant**
**P HR 95% CI**	**P**	
0.044 2.9 1-8.3	0.022	miR-192 expression (high vs. low)
0.001 12 2.7-53	0.0001	Risk (high vs. low)
NS	0.033	MycN (amplified vs. non-amplified)
NS	0.001	Stage (3,4 vs. 1,2,4S)

HR: hazard ratio; CI: confidence interval

Moreover, a significant inverse correlation between miR-192 expression levels and Dicer1 was detected in 21 primary NB samples in which the results of both analyzes were available (p=0.018). Thus, we decided to focus on miR-192 and to study its affect on Dicer1 and cell proliferation in NB.

### miR-192 regulates Dicer1 mRNA and protein in NB cell lines

**Figure 4:**
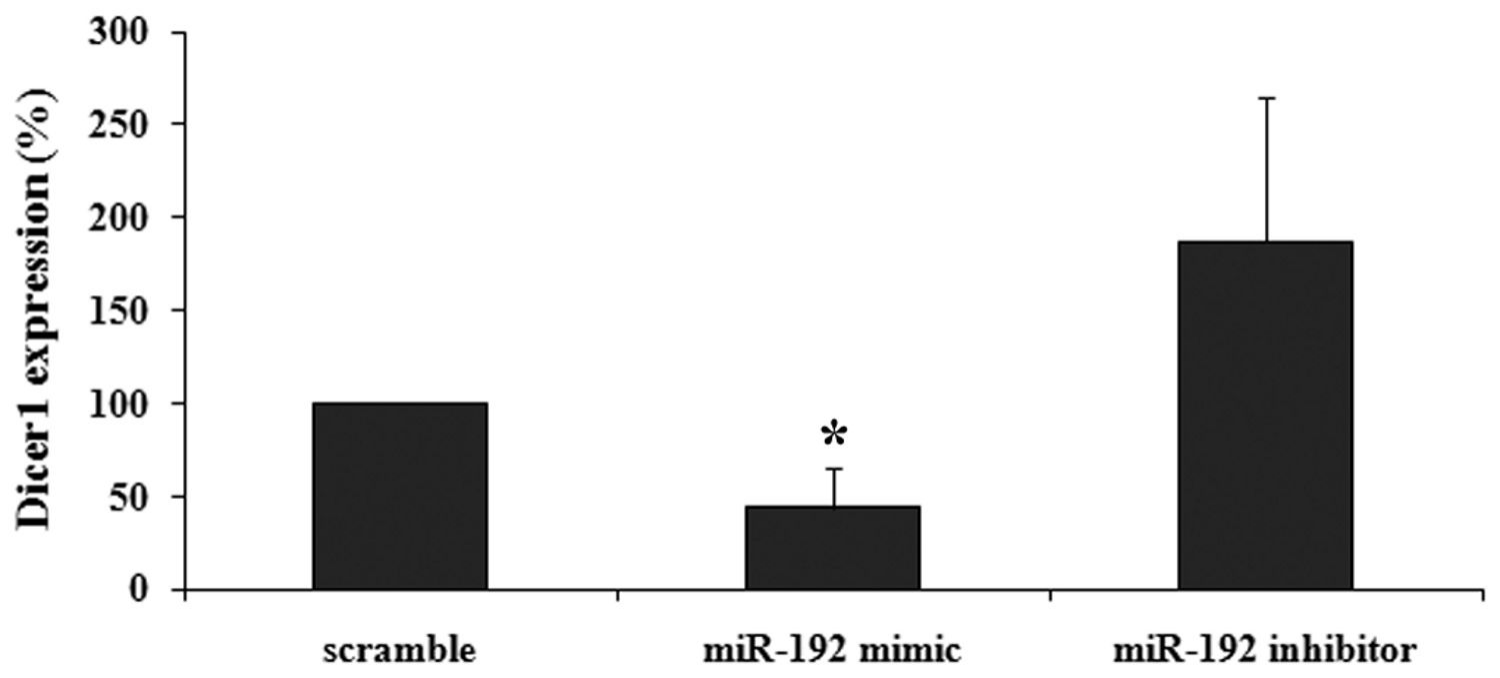
miR-192 regulates Dicer1 mRNA expression. Following transfection of miR-192 mimic and inhibitor, a significant decrease (*p=0.028) or elevated Dicer1 mRNA expression levels were detected, respectively.

**Figure 5:**
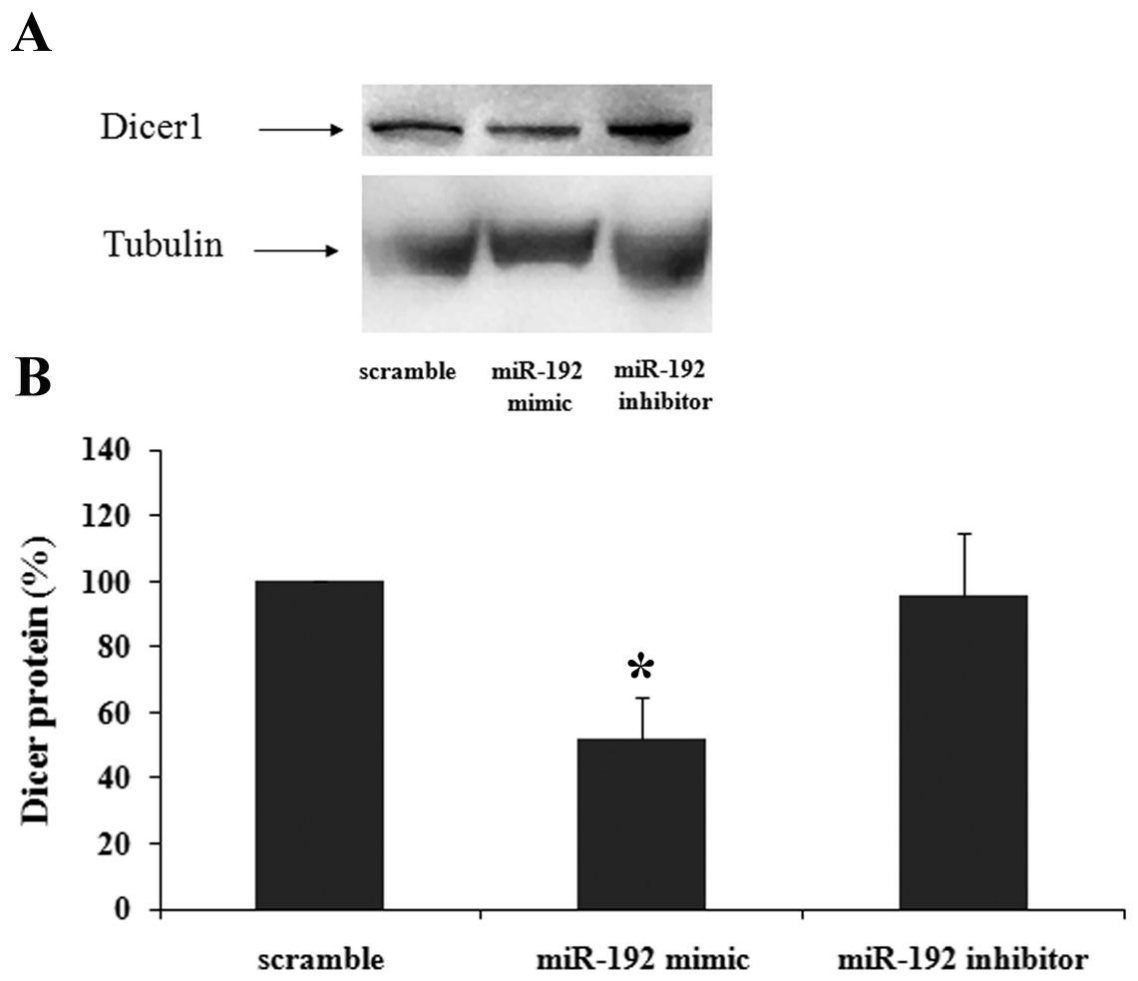
miR-192 regulates Dicer1 protein expression. A. Dicer1 protein expression in NUB6 cells, as evaluated by Western blotting, after exposure to miR-192. B. Graphic illustration of relative Dicer1 protein levels (normalized to tubulin) in Nub6 cells following transfection with miR-192 mimic (*p=0.04) and inhibitor. Data are presented as means ± SE of three independent experiments.

Dicer1 mRNA and protein expression levels were measured following transfection of miR-192 mimic or inhibitor into NUB6 cell line. Following overexpression of miR-192, we identified a significant decrease in Dicer1 mRNA expression level (56%, p=0.028), while silencing of miR-192 correlated with elevated expression of Dicer1 mRNA, although not-significant (Figure 4). Overexpression of miR-192 caused a significant reduction in the protein expression of Dicer1 (51%, p=0.04) detected by Western blotting, as seen in Figure 5A and 5B. Nub6 cells transfected with miR-192 inhibitor expressed equivalent levels of Dicer1 protein and a negative control. 

### Dicer1 is a direct target of miR-192

Computional analysis predicted three BSs for miR-192 in the 3'UTR of Dicer1. We defined these BSs on the 3' UTR of pIS1 DICER1 long UTR as: BS1 on position 1232-1238, BS2 on position 2282-2288; BS3 on position 3049-3055.

We designed four different plasmid constructs (Figure 1). The first plasmid contained mutations in all three BSs (ALL MUT).The second plasmid contained two mutations in BS2 and BS3, leaving BS1 active; the third plasmid consisted of mutations in BS1 and BS3, leaving BS2 active. The fourth plasmid included mutations in BS1 and BS2, leaving BS3 active

**Figure 6:**
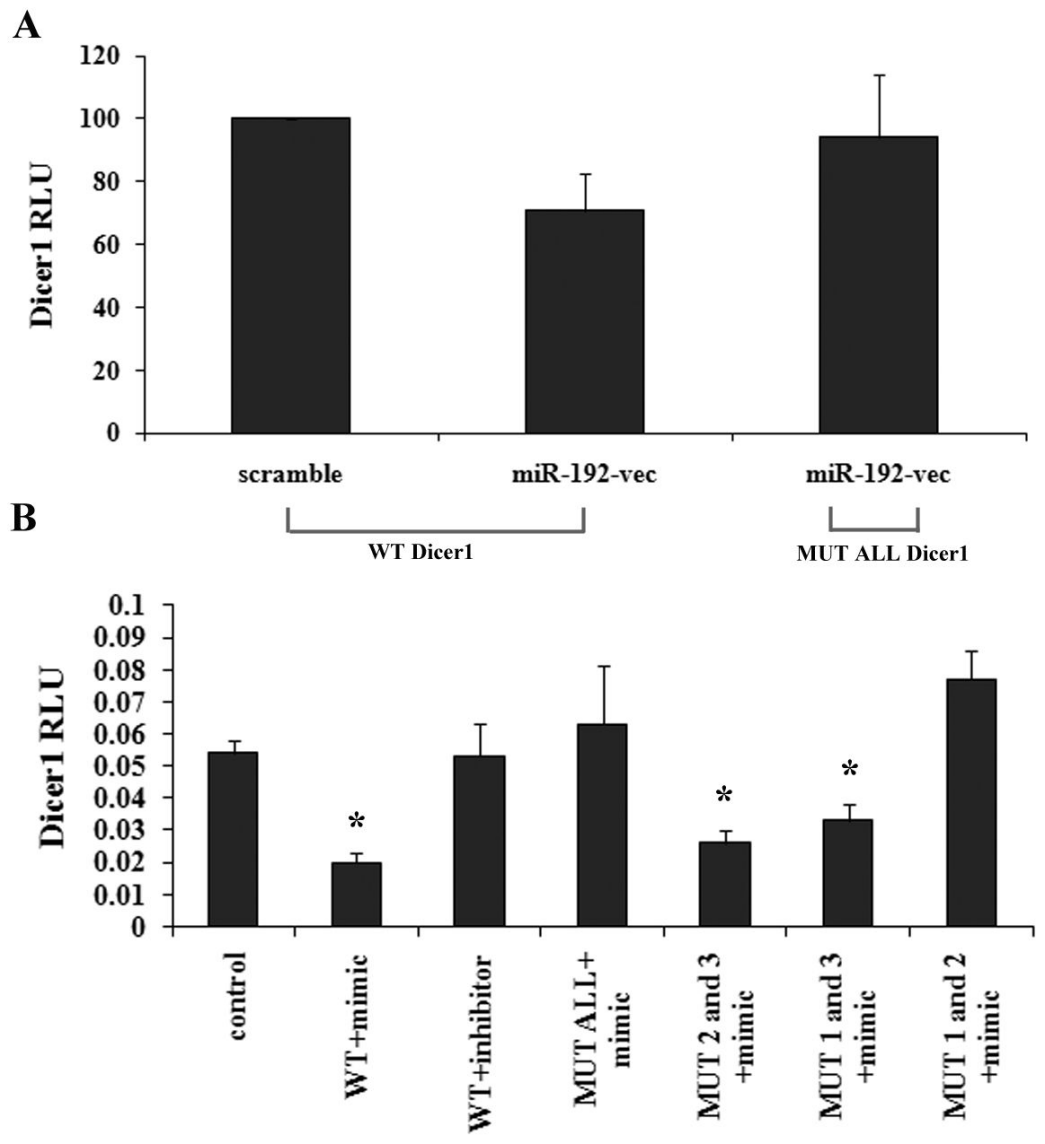
3′UTR of Dicer 1 is directly targeted by miR-192. The dual luciferase assay detected that Dicer1 is modulated by miR-192 in NB cell lines. The relative luciferase unit (RLU) was measured in SHEP (A) or NUB (B) cells. A. The dual-luciferase assay resulted in a significant reduction of RLU of WT Dicer1 (3' UTR of Dicer1 wild type) following transfection with miR-192-vec (*p=0.049). B. Following transfection with miR-192 mimic, WT Dicer1 RLU was significantly decreased (*p=0.0003). Following mutagenesis, cells were transfected with Dicer1 plasmid in which mutations were introduced in all three BSs of Dicer1 (MUT ALL); active BS1 (mutated at BS2+BS3)(* p=0.004); active BS2 (mutated at BS1+BS3) (* p=0.04) and active BS3 (mutated at BS1+BS2). Values are expressed as the mean ± SE of combined results from three independent experiments.

To validate the relative relevance of each one of the potential BSs of miR-192 in the 3′ untranslated region (3′UTR) of Dicer1, two different dual-luciferase reporter assays were carried out in NB cell lines. The dual-luciferase assay resulted in a significant reduction of 30% in the relative luciferase unit (RLU) of Dicer1 as was evident in SHEP cell line (non-MYCNA) transfected with miR-192-vec (p=0.049; Figure 6A). Moreover, a significant decrease of 59% in the level of Dicer1 RLU was evident in the NUB6 cell line (MYCNA) after transfection with the miR-192 mimic (p=0.0003; Figure 6B). Following transfection with miR-192 inhibitor the Dicer1 RLU returned to the control basal level (determined by transfection of scrambled miR sequences). These results suggest that miR-192 expression regulates Dicer1 protein expression.

When introducing the plasmid harboring mutations in all three BSs (ALL MUT) into both cells lines, the expression levels of Dicer1 were comparable to baseline. Following transfection with the second plasmid that contained active BS1 (mutations in BS2 and BS3), we identified a significant decrease of 53% (p=0.004) in Dicer1 RLU. Similarly, following transfection with the third plasmid with active BS2 (mutated in both BS1 and BS3), a significant decrease of 42% (p=0.04) in Dicer1 RLU was detected. On the other hand, following transfection of the fourth plasmid that included active BS3 (mutations in BS1 and BS2), the expression of Dicer1 was not reduced but non-significantly increased (38%) (Figure 6B). Taken together, these results confirm that Dicer1 is directly regulated by miR-192. These results indicate that miR-192 binds and regulates Dicer1 through BS1 and BS2.

### miR-192 effect on NB cell viability, cell proliferation and migration capability

**Figure 7:**
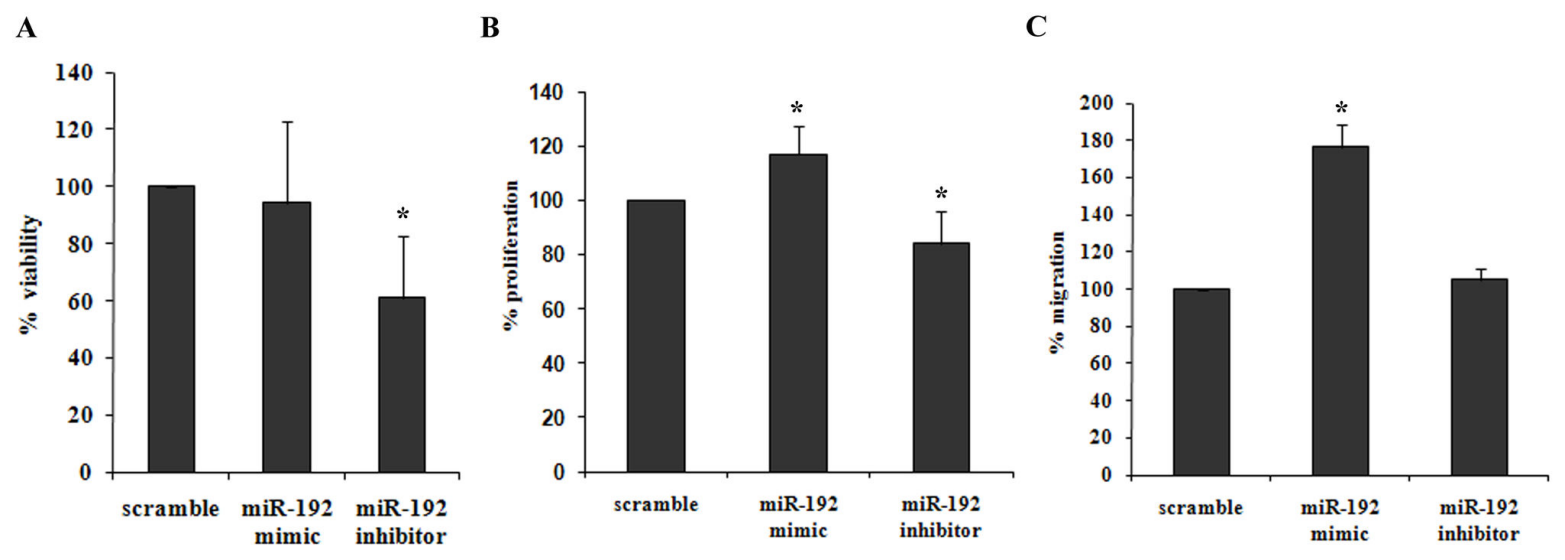
miR-192 is associated with NB cell viability, proliferation and migration capability. Following transfection of miR-192 mimic and inhibitor, we measured the viability of NUB6 cells by direct counting (A), proliferation properties of the cells by XTT analysis (B) and the migration ability of NUB6 cells (C). A. Inhibition of miR-192 resulted in significantly decreased cell viability (*p=0.042). B. Following transfection of miR-192 mimic and inhibitor, a significant increase(*p=0.044) or decrease (*p=0.038) was detected in the proliferation rate respectively. C. Overexpressing miR-192 resulted in a significant increase in cell migration (*p= 0.00036). Values are expressed as the mean ± SD of combined results of three independent experiments.

Next, we were interested in investigating the affect of miR-192 on the aggressiveness of NB cells. NUB6 cells transfected with miR-192-inhibitor led to a 39% decrease in cell viability (p=0.042, Figure 7A). NUB6 cells transfected with miR-192-mimic and inhibitor led to an increase of 17% in proliferation (p=0.044) or a significant decrease of 16% in the proliferation rate (p=0.038, Figure 7B), respectively. Over-expressing miR-192 exhibited a significantly increased migration ability (a 1.7-fold increase, p= 0.00036) as compared to the negative control (Figure 7C). 

## Discussion

The expression of Dicer1 is altered in various adult malignancies. Low Dicer1 expression is significantly associated with poor prognosis in ovarian cancer and lung adenocarcinoma [7,9,10]. 

Dicer1 plays an important role in neuronal development [11-13]. Dicer1 may be important in NB tumor initiation, since NB is a childhood cancer arising from the sympaticoadrenal lineage of the neural crest. Previously, low expression levels of Dicer1 were found to correlate with a poor outcome in NB patients and to be an independent prognostic marker in patients with non-MYCNA tumors [6]. We also demonstrated that Dicer1 correlates with a poor prognosis in NB. 

In view of these studies we were interested in searching for mechanisms that regulate Dicer1 expression. One such mechanism could be a change in copy number due to chromosomal alterations. For example, high levels of Drosha were detected in advanced cervical squamous cell carcinoma due to the gain of chromosome 5p [10,14]. It is known that 22% of primary NB tumors harbor tumor deletion of chromosome 14q23-32, which includes the Dicer1 locus [9,10,14]. Our results do not support that the 14q23-32 deletion as a mechanism for the down-regulation of Dicer1 in NB tumors, since only 5% of tumors exhibited deletions. Specific miRs might also be a mechanism for Dicer1 down-regulation. We measured the expression levels of several predicted miRs that target Dicer1. These miRs were chosen on the basis of their involvment in NB (for example, miR-124 and miR-125b) [15-17] and/ or they have additional predicted target genes that seem to be relevant for the disease (such as ALK, Slug and Skp2) [18].

Of the miRs studied, a significant association between poor outcome and high levels of miR-192 was observed, suggesting the important role of miR-192 in tumor progression in NB. Similar to our results, miR-192 was over-expressed in the adenocarcinoma of the esophagus [19], in peripheral lung adenocarcinoma, in carcinomas that frequently metastasize to lung pleura [20] and in colorectal adenocarcinomas [21]. We identified, for the first time, a significant correlation between low Dicer1 protein levels and high miR-192 levels, suggesting that Dicer1 is a target of miR-192 and we have provided evidence that miR-192 directly binds Dicer1 independently of MYCN status. 

The 3'UTR of Dicer1 was predicted to harbor three BSs of miR-192. Significantly, a potent inhibition of luciferase activity was mediated by the 3'UTR of Dicer1 in NUB6 and SHEP cells ectopically expressing miR-192. In contrast, no significant change in Dicer1 levels was detected by a construct mutated at all three miR-192-predicted target sites or in cells in which miR-192 expression was silenced. Our results indicate that modulation of miR-192 expression may play a role in Dicer1 protein expression. Down-regulation of Dicer1 was detected in NB cells expressing high levels of miR-192. Following site directed mutagenesis; we were able to show that miR-192 binds and regulates Dicer1 through BS1 and BS2 of the 3'UTR of Dicer1.

Thus, we confirmed that miR-192 directly targets Dicer1 expression in NB and can be added to the list of miRs that regulate Dicer1 such as let-7 and miR-103/107 [21-23 ].

Overexpression of miR-192 resulted in a significant increase in cell proliferation and in the migration ability of NB cells, while inhibition of miR-192 correlated with elevated expression of Dicer1 mRNA and caused a significant decrease in both the proliferation rate and in cell viability. Similarly, in HeLa cells, inhibition of miR-192 caused a decrease in cell growth [24]. 

Several studies imply that Dicer1 expression is an important player in the metastatic process [22,25]. We hypothesised that the adverse effect of Dicer1 may be, in part, as a result of miR-192 regulation.

To summarize, we hereby show that miR-192 is an independent prognostic marker for relapse in NB and that low Dicer1 expression correlates with a poor outcome. We have demonstrated that miR-192 directly regulates Dicer1 in NB through the BS on position 1232-1238 and BS on position 2282-2288 of 3'UTR Dicer1, regardless of MYCN status. Further studies are warranted to establish the function of miR-192 and Dicer1 in NB tumor progression.
